# Survival after cardiopulmonary arrest due to Capnocytophaga canimorsus sepsis: A case successfully managed using plasma exchange

**DOI:** 10.1016/j.ijregi.2025.100826

**Published:** 2025-12-19

**Authors:** Yoshio Nakano, Hiroka Serizawa, Iwao Gohma

**Affiliations:** Department of Respiratory Medicine, Sakai City Medical Center, Sakai, Japan

**Keywords:** Capnocytophaga canimorsus, Sepsis, Cardiopulmonary arrest, Plasma exchange, Thrombotic microangiopathy, Critical care

## Abstract

Capnocytophaga canimorsus sepsis is potentially fatal, even in immunocompetent hosts. This report describes a case involving a 57-year-old woman, who developed fever after a dog bite and initially presented without an evident focus. On the day of admission, she deteriorated to pulseless electrical activity during transport and achieved return of spontaneous circulation after approximately 23 minutes of resuscitation. Broad-spectrum antimicrobials (meropenem) were initiated for septic shock, and Capnocytophaga canimorsus subsequently grew in both the pre-admission and admission blood cultures. Peripheral discoloration suggested purpura fulminans. A precipitous decline in platelet count, schistocytosis, elevated lactate dehydrogenase levels, and acute kidney injury fulfilled the criteria for thrombotic microangiopathy, whereas ADAMTS13 activity was normal. Intensive care consisted of red blood cell and platelet transfusions, continuous hemodiafiltration transitioning to intermittent hemodialysis, mechanical ventilation with tracheostomy, and therapeutic plasma exchange once daily for three sessions. Hematological abnormalities resolved, schistocytes disappeared, organ dysfunction improved, and she was ultimately removed from ventilation and dialysis with full neurological recovery. This report describes survival after cardiopulmonary arrest due to Capnocytophaga canimorsus sepsis and suggests that plasma exchange may be a useful adjunct for secondary thrombotic microangiopathy in this setting. Early diagnosis, prompt antibiotic treatment, and multidisciplinary critical care are essential.

## Highlights

• First reported survival after cardiopulmonary arrest caused by Capnocytophaga canimorsus sepsis.
• Plasma exchange contributed to recovery from secondary thrombotic microangiopathy.
• Early diagnosis and multidisciplinary intensive care were crucial for full recovery.

## Introduction

Capnocytophaga canimorsus (C. Canimorsus) infection is a bacterial disease that typically follows a dog bite, with the earliest case of sepsis reported in the United States in 1976 [1]. Although severe illness has been described predominantly in immunocompromised hosts [2], it can also occur in individuals with normal immune status [3,4]. When C. canimorsus infection progresses to sepsis, the mortality rate is high [3]. In severe presentations, patients may develop purpura fulminans, disseminated intravascular coagulation (DIC), and thrombotic microangiopathy (TMA), and there are case reports describing recovery from C. canimorsus-associated TMA following plasma exchange (PE) [5]. Despite numerous reports of severe disease, no published cases have documented survival after cardiopulmonary arrest (CPA) caused by C. canimorsus, and few reports have demonstrated the therapeutic efficacy of PE for C. canimorsus-associated TMA [5]. Herein, we describe a case of C. canimorsus infection in a patient without underlying comorbidities who experienced CPA, was successfully resuscitated, and ultimately survived with intensive care, including PE.

## Case presentation

A 57-year-old woman with a history of depression and an otherwise normal immune status maintained a 3-year-old dog (Shiba Inu). Six days before admission (day 6), the patient had sustained a bite from this dog on her right fourth finger. Two days before admission (day 2), fever developed; 1 day before admission (day 1), she presented to the emergency department with fever but no clear infectious focus. Vital signs were stable, and laboratory investigations revealed a white blood cell count of 7760/µl and a C-reactive protein level of 0.29 mg/dl. The patient was discharged after blood cultures were obtained.

On admission (day 1), emergency medical services were provided for recurrent fever and impaired consciousness. During ambulance transport, she developed pulseless electrical activity. Cardiopulmonary resuscitation was initiated, and return of spontaneous circulation was achieved after administration of two ampoules of epinephrine, with a maximal arrest time of 23 minutes. The patient was subsequently admitted to the intensive care unit. Blood cultures drawn on day 1 and day 1 later revealed C. canimorsus. Positive blood culture bottles were subcultured onto blood agar plates and chocolate agar, and bacterial growth was observed on both media. Species-level identification of the isolate was achieved using matrix-assisted laser desorption/ionization time-of-flight mass spectrometry (MALDI-TOF MS). Broad-spectrum antimicrobial therapy with meropenem was initiated for the sepsis/septic shock. On arrival, peripheral discoloration of the extremities suggested purpura fulminans.

**Figure 1:**
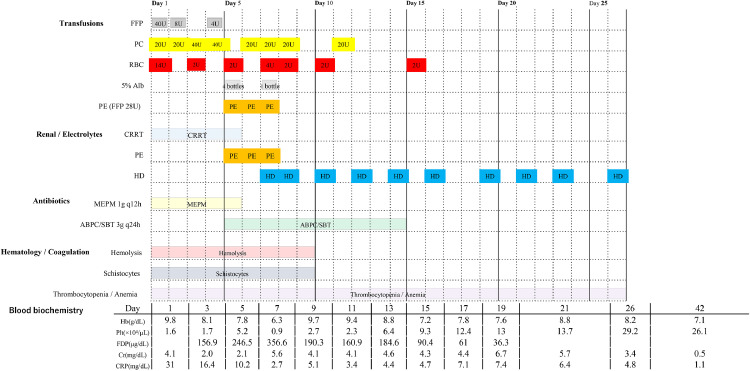
Clinical course. Timeline of key interventions and labs after admission. Rows (top to bottom): transfusions (FFP, PC, RBC, 5% albumin, PE), renal support (CRRT/HD), antibiotics (meropenem → ampicillin/sulbactam), hematology/coagulation (hemolysis, schistocytes, thrombocytopenia/anemia), and blood biochemistry trends. Horizontal bars show duration; numbers indicate daily dose or schedule (e.g., units/bottles, q12h). ABPC/SBT, ampicillin/sulbactam; Cr, serum creatinine; CRP, C-reactive protein; CRRT, continuous renal replacement therapy; FDP, fibrin/fibrinogen degradation products; FFP, fresh frozen plasma; Hb, hemoglobin; HD, hemodialysis; MEPM, meropenem; PC, platelet concentrates; PE, therapeutic plasma exchange; Plt, platelet count; q12h/q24h, every 12 hours/every 24 hours; RBC, red blood cells (packed); 5% Alb, 5% albumin; schistocytes, fragmented red cells; thrombocytopenia, low platelet count; anemia, low hemoglobin; blood biochemistry, serum chemistry tests; hemolysis, red cell destruction.

During hospitalization, she developed a precipitous decline in platelet count, schistocytosis, elevated lactate dehydrogenase levels, and worsening renal function, fulfilling the criteria for thrombotic microangiopathy. The patient underwent multiple red blood cell and platelet transfusions, and therapeutic PE was performed three times, after which the schistocytes disappeared and thrombocytopenia improved. The acute kidney injury was initially anuric; continuous hemodiafiltration was initiated due to hemodynamic instability and later transitioned to intermittent hemodialysis once stable. Prolonged mechanical ventilation was required, and a tracheostomy was performed on hospital day 12. She was transferred out of the intensive care unit on day 19 of hospitalization with ongoing dialysis. Subsequently, tracheostomy and dialysis were discontinued, and her level of consciousness returned to normal. Rehabilitation for deconditioning and local care of distal necrotic lesions is ongoing. On day 76, the patient was transferred to a rehabilitation facility for rehabilitative care (Figure 1).

## Discussion

Herein, we report a case of C. canimorsus infection in which the patient was successfully resuscitated from CPA and ultimately survived in intensive care. This case highlights two key points. First, recovery is achievable even after CPA due to C. canimorsus infection and, second, PE may have therapeutic utility for C. canimorsus-associated TMA.

Although C. canimorsus infection is classically associated with severe sepsis and high mortality in individuals with alcohol use disorder(s) or a history of splenectomy, approximately 40% of sepsis cases occur in patients without predisposing conditions [6]. In immunocompetent hosts, 47.7% of sepsis cases require hospitalization, with an overall case fatality rate of 29.7% and a particularly high mortality rate of 55.7% among those with sepsis [3]. In the case described herein, a previously healthy, immunocompetent patient developed septic shock due to C. canimorsus that progressed to CPA on arrival. To the best of our knowledge, no previous reports have documented survival after CPA caused by C. canimorsus infection. Despite the severity of the illness, complicated by post-resuscitation TMA and acute kidney injury requiring intensive care, the patient survived, suggesting that even post-arrest C. canimorsus sepsis can be managed with timely and appropriate critical care.

The second notable feature is the probable benefit of PE for TMA that develops after resuscitation. TMA is characterized by the triad of thrombocytopenia, microangiopathic hemolytic anemia, and organ injury caused by platelet-rich microthrombi [7]. In severe C. canimorsus sepsis, secondary TMA can occur in addition to DIC [5,8]. Although our patient was initially managed for DIC, progressive thrombocytopenia, appearance of schistocytes, and renal dysfunction supported the diagnosis of TMA. The etiology of TMA includes thrombotic thrombocytopenic purpura, typical and atypical hemolytic uremic syndromes, and secondary forms triggered by infections, malignancies, and autoimmune diseases. In this case, ADAMTS13 activity was normal, and inhibitors were absent, supporting infection-associated secondary TMA. Similar findings, including normal ADAMTS13 activity and the absence of inhibitors, have been reported for TMA secondary to C. canimorsus infection [5].

In the intensive care setting, PE has been associated with improved outcomes in patients with TMA. Darmon *et al.* [9] analyzed data from 36 intensive care unit patients with TMA and found that those treated with PE experienced faster organ recovery and significantly lower mortality, supporting the clinical utility of PE in severe TMA. Conversely, Kaname *et al.* [10] examined outcomes of PE for trigger-associated TMA and reported limited response rates (5-63%) with substantial mortality and relapse, suggesting that PE alone may be insufficient in a subset of patients and that additional therapies may be necessary. Reports specifically addressing PE for TMA complicated by C. canimorsus infection remain scarce; however, Tani *et al.* [5] reviewed 11 cases (including their own) and noted that many patients underwent PE with favorable outcomes.

In our patient, TMA secondary to C. canimorsus infection developed after resuscitation. Alongside antimicrobial therapy and supportive transfusions, we performed PE once daily for 3 consecutive days, after which hematological parameters and organ dysfunction gradually improved. These observations indicate that PE should be considered a potential adjunctive therapy for C. canimorsus-associated secondary TMA in combination with prompt infection control and comprehensive critical care.

## Conclusion

The patient described experienced CPA due to C. canimorsus infection, but was successfully treated through intensive care that included PE. This case suggests that even patients who experience post-arrest C. canimorsus sepsis can survive with appropriate management, and that PE may have a role in the treatment of secondary TMA associated with this infection.

## Author contributions

Yoshio Nakano was responsible for study conception, data collection, and manuscript preparation. Hiroka Serizawa and Iwao Gohma reviewed and revised the manuscript. All authors approved the final manuscript.

## Declaration of generative AI and AI-assisted technologies in the manuscript preparation process

During the preparation of this work, the authors used *ChatGPT (OpenAI)* to improve the clarity and fluency of the English language. After using this tool, the authors carefully reviewed and edited the text to ensure that the content represents their own original work, analysis, and interpretation. The authors take full responsibility for the content of this publication.

## Funding

This research did not receive any specific grant from funding agencies in the public, commercial, or not-for-profit sectors.

## Ethical approval

Written informed consent for publication of this manuscript and accompanying images was obtained from the patient and her family in accordance with institutional requirements.

## Declaration of competing interest

The authors have no competing interests to declare.
